# Application of the Interaction between Tissue Immunohistochemistry Staining and Clinicopathological Factors for Evaluating the Risk of Oral Cancer Progression by Hierarchical Clustering Analysis: A Case-Control Study in a Taiwanese Population

**DOI:** 10.3390/diagnostics11060925

**Published:** 2021-05-21

**Authors:** Hui-Ching Wang, Meng-Chun Chou, Chun-Chieh Wu, Leong-Perng Chan, Sin-Hua Moi, Mei-Ren Pan, Ta-Chih Liu, Cheng-Hong Yang

**Affiliations:** 1 Graduate Institute of Clinical Medicine, College of Medicine, Kaohsiung Medical University, Kaohsiung 807, Taiwan; joellewang66@gmail.com; 2 Division of Hematology and Oncology, Department of Internal Medicine, Kaohsiung Medical University Hospital, Kaohsiung Medical University, Kaohsiung 807, Taiwan; 3 Faculty of Medicine, College of Medicine, Kaohsiung Medical University, Kaohsiung 807, Taiwan; oleon24@yahoo.com.tw; 4 Drug Development and Value Creation Research Center, Kaohsiung Medical University, Kaohsiung 807, Taiwan; lazzz.wu@gmail.com; 5 Department of Nursing, Kaohsiung Medical University Hospital, Kaohsiung Medical University, Kaohsiung 807, Taiwan; bulemarymog5681@gmail.com; 6 Department of Pathology, Kaohsiung Medical University Hospital, Kaohsiung Medical University, Kaohsiung 807, Taiwan; 7 Department of Otolaryngology-Head and Neck Surgery, Kaohsiung Medical University Hospital, Kaohsiung Medical University, Kaohsiung 807, Taiwan; 8 Department of Otorhinolaryngology-Head and Neck Surgery, Kaohsiung Municipal Ta-Tung Hospital and Kaohsiung Medical University Hospital, Kaohsiung 807, Taiwan; 9 Center of Cancer Program Development, E-Da Cancer Hospital, I-Shou University, Kaohsiung 824, Taiwan; moi9009@gmail.com; 10 Department of Hematology-Oncology, Chang Bing Show Chwan Memorial Hospital, Changhua 505, Taiwan; 11 Department of Electronic Engineering, National Kaohsiung University of Science and Technology, Kaohsiung 807, Taiwan; 12 Ph. D. Program in Biomedical Engineering, Kaohsiung Medical University, Kaohsiung 807, Taiwan

**Keywords:** oral cancer, risk stratification, progression-free survival

## Abstract

The aim of this single-center case-control study is to investigate the feasibility and accuracy of oral cancer protein risk stratification (OCPRS) to analyze the risk of cancer progression. All patients diagnosed with oral cancer in Taiwan, between 2012 and 2014, and who underwent surgical intervention were selected for the study. The tissue was further processed for immunohistochemistry (IHC) for 21 target proteins. Analyses were performed using the results of IHC staining, clinicopathological characteristics, and survival outcomes. Novel stratifications with a hierarchical clustering approach and combinations were applied using the Cox proportional hazard regression model. Of the 163 participants recruited, 102 patients were analyzed, and OCPRS successfully identified patients with different progression-free survival (PFS) profiles in high-risk (53 subjects) versus low-risk (49 subjects) groups (*p* = 0.012). OCPRS was composed of cytoplasmic PLK1, phosphoMet, and SGK2 IHC staining. After controlling for the influence of clinicopathological features, high-risk patients were 2.33 times more likely to experience cancer progression than low-risk patients (*p* = 0.020). In the multivariate model, patients with extranodal extension (HR = 2.66, *p* = 0.045) demonstrated a significantly increased risk for disease progression. Risk stratification with OCPRS provided distinct PFS groups for patients with oral cancer after surgical intervention. OCPRS appears suitable for routine clinical use for progression and prognosis estimation.

## 1. Introduction

Oral squamous cell cancer (OSCC) is the sixth most common cancer worldwide, with 630,000 new cases and 350,000 deaths estimated annually [1]. Its striking worldwide incidence and socioeconomic burden encourage extensive research on factors that could modify clinical outcomes [2]. Despite multidisciplinary interventions, a high incidence of oral cancer recurrence and metastasis affects the quality of life and survival in patients [3,4]. Therefore, it remains crucial to identify prognostic biomarkers and risk stratifications for improved disease management.

Reported and well-known risk factors for oral cancer include alcohol consumption, betel nut use, and cigarette smoking [5,6]. Several predictive factors, including age, ethnicity, gender, primary site, grade, and therapy, have demonstrated associations between these sociodemographic factors and survival in oral and pharyngeal carcinoma [2]. In clinical practice, the tumor-node-metastasis (TNM) system is the most prevalent tool for prognostic evaluation of OSCC. However, this system lacks immediacy and convenience, necessitating extensive physical and imaging examinations. Furthermore, biological phenotypes and clinical presentations differ even at identical diagnostic stages. More accurate and timely prognostic biomarkers are requisite for OSCC, especially in Asian populations.

Reportedly, DNA repair gene XRCC1 polymorphisms could alter the activity of the XRCC1 protein, leading to defective DNA repair and influencing p53 gene mutation, which has demonstrated a negative impact in Taiwanese patients with OSCC [7]. In Korea, Choi et al. have reported that patterns with single nucleotide polymorphisms in ECRG1 and FGFR4 genes were associated with the clinical nodal status. The FGFR4 Arg allele carrier correlated with advanced nodal stage when compared with the Gly allele [8]. Conversely, positive prognostic markers have been reported, including APOBEC3A. In Taiwanese data, high APOBEC3A expression, especially among APOBEC3B-deletion alleles, has been associated with better overall survival (OS) [9]. Capillary electrophoresis-mass spectrometry (CE-MS) metabolome analysis of saliva samples has been performed in Japanese patients with OSCC, and 25 metabolites have been identified as potential markers that could be used to distinguish between patients with OSCC and healthy controls [10]. Proteins encoded by *EGFR, TP53, CCND1,* and *RB1* are associated with OSCC progression [11,12,13]. In a study evaluating 55 patients regularly using betel nut, immunohistochemistry (IHC) of cyclin D1, MDM2, and γ-catenin has revealed their prognostic potential in buccal squamous cell carcinoma (SCC) [11]. In United States, a similar report demonstrated that APE1, as the DNA repair and redox gene regulator, served as a potential prognostic signature that identifies patients with worsened survival [14]. In Brazil, the expressions of DNA nucleotide repair proteins, TFIIH and XPF, had a potential value for predicting the progression of tongue cancer patients [15]. DNA mismatch repair deficiency in Australian patients with oral cancer was associated with more advanced primary tumors [16]. However, genomic and molecular profiles of OSCC to guide clinical medicine remain scarce.

Hierarchical agglomerative clustering analysis has demonstrated a rapid and invaluable strategy to manage high-dimensional datasets [17], demonstrating the ability to simultaneously dissect substantial data with multiple layers of the clustering structure. Hierarchical clustering algorithms have been applied for exploratory analysis of gene expression data [18]. It is highly sensitive to background noise and can recognize interactions between factors by considering the similarity within a cluster and dissimilarity between clusters [19]. Previously, hierarchical agglomerative clustering algorithms have been successfully applied to the functional grouping of biological data [20,21]. These methods have been used to identify important clinical features in various cancers, including lung and breast cancers [22,23]. However, hierarchy results derived from the clustering algorithm are generally difficult to apply to clinical settings. Therefore, corresponding risk modules based on the agglomerative clustering results are necessary to generalize the findings for clinical applications.

Reportedly, some well-established IHC markers provide useful prognostic and predictive information in addition to classical clinical factors [24]; however, most have not been validated for clinical use. The Cancer Genome Atlas demonstrated a comprehensive landscape of somatic genomic alterations for head and neck cancer [25]. Similarly, Gene Expression Omnibus (GEO) database also provided a high throughput platform for recognition of more potential predictors [26]. Molecular methods with DNA amplifications scattered from 8q22.2 to 8q24.3 is a candidate molecular signature associated with poor prognosis in OSCC patients [27]. Further patient-tailored identification of biomarkers and therapeutic candidate alteration is an important issue that needs to be faced up. Synthetic lethality (SL) seems to play an important role in oral cancer for the promising results of antineoplastic agents, poly (ADP-ribose) polymerases (PARP) inhibitors [28,29]. The primary aim of this study was to stratify different patient risks based on newly discovered IHC markers associated with synthetic lethality using a hierarchical clustering approach, providing the best prognostic information for patients with OSCC. We analyzed the IHC data and clinicopathological and prognostic features in Taiwanese patients with OSCC using our study cohort. Finally, to classify patients and obtain an overall prediction model, we generated a prognostic model integrating oral cancer protein risk stratification (OCPRS) with the most significant IHC markers.

## 2. Materials and Methods

### 2.1. Data Set

This study was approved by the Institutional Review Board and Ethics Committee of Kaohsiung Medical University Hospital (KMUHIRB-E(I)-20170034, approved on 10 March 2017). The data were analyzed anonymously, and therefore, no informed consent was required. All methods were performed under approved guidelines and regulations. We collected 163 cases of oral cavity cancers from the Kaohsiung Medical University Hospital with a 5-year follow-up. The inclusion criteria were: 20 years of age or older at diagnosis, histology of SCC with grade 1 to grade 3, ICD-9 site code specific for the oral cavity, patients who underwent surgical interventions, and diagnosis between 2012 and 2014. The exclusion criteria included: patients who underwent biopsy without surgical intervention, with secondary malignancy, tumor histology of carcinoma in situ, and SCC from the nasopharynx, oropharynx, hypopharynx, and larynx. Histological grades were defined as grade 1, well differentiated; grade 2, moderately differentiated; grade 3, poorly differentiated. We collected medical and demographic data, including age, gender, alcohol consumption, betel nut usage, tobacco habits, and other clinical parameters, retrospectively from the medical records or during patient interviews. The clinicopathological factors included histologic type and grade, tumor size, lymph node status, surgical margin, perineural invasion (PNI), lymphovascular invasion (LVI), and extranodal extension (ENE). Patients without complete clinical data and clinicopathological factors were excluded, and 102 patients were analyzed. We evaluated the results of a retrospective study with the primary endpoint of assessing outcomes at a comprehensive cancer institution in southern Taiwan. We analyzed the OS and progression-free survival (PFS) (defined as the time from registration to objective disease progression or death from any cause) after surgical intervention.

### 2.2. Computation of Gene Expression Profiles for Oral Cavity Cancer versus Non-Cancerous Tissues

The approach to successfully find out novel IHC prognostic markers associated with synthetic lethality in colorectal cancer and lung adenocarcinoma was adopted in our study [30,31]. As previous studies, we selected a list of SL-associated genes, including several oncogenes, tumor-suppressor genes, and genome stability genes. From these validated SL-associated genes, twenty-one genes were used for IHC staining at different cellular locations. We combined the associated 32 individual IHC expressions and identified novel IHC prognostic markers among them.

**Figure 1:**
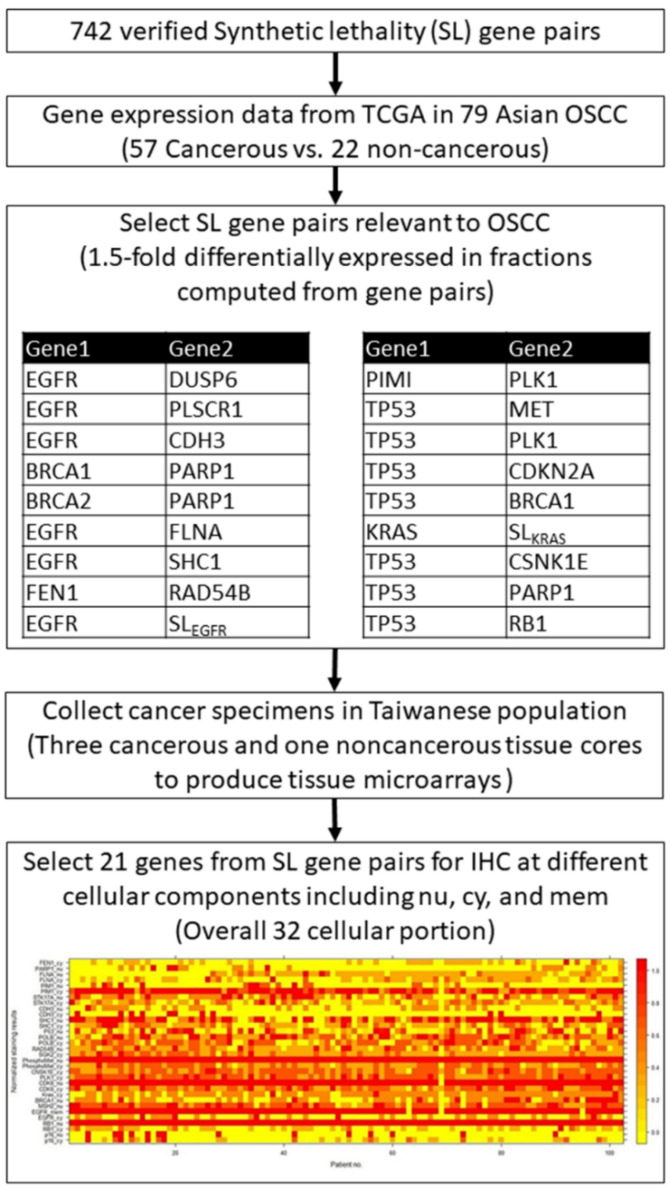
Study flowchart and the heatmap of protein staining matrix according to 32 individual IHC results.

Figure 1 illustrates the study workflow for target genes selection from the validated SL gene pairs and the yield of protein staining matrix according to the 32 individual IHC. First, we selected 742 SL pairs relevant to OSCC, and obtained the microarray gene expression data from the cancer genome atlas (TCGA) of 79 Asian OSCC. Gene expression datasets were screened according to the following parameters: cancerous and noncancerous tissues, no treatments, no metastasis, and Affymetrix chips (up to November 2010). OSCC genes were downloaded from the GEO database [26]. Gene expression data were collected from patients of Han Chinese origin (57 OSCC and 22 noncancerous tissues from Taiwanese patients, GSE 25099), the same ethnicity as that of IHC and clinicopathological data used previously [27]. Gene expression profiles for the 57 OSCC and 22 noncancerous tissues in the dataset were quantile-normalized using “expresso” in R, and log ratios were computed for the target gene expression in each cancerous tissue versus the mean expression in the noncancerous tissues. The selected SL gene pairs were further sorted by the fractions of the upregulation and downregulation patterns, and the SL pairs with 1.5-fold differentially expressed in fractions computed from gene pairs were selected as target genes. Overall, 21 genes were selected using the above criteria, and the cancer specimen collected from the Taiwanese population in the current study were then used to produce tissue microarrays with three cancerous and one noncancerous tissue cores as our previous study [32]. The tissue microarrays were further processed for IHC for 21 target proteins in different cellular components including nucleus (nu), cytoplasm (cy), and membrane (mem). Hence, a total of 32 protein staining scores were obtained from the 21 target proteins.

### 2.3. Protein Staining

Representative sections of the hematoxylin and eosin (H&E)-stained biopsy-confirmed tissues of the 102 patients with OSCC were selected by pathologists (Chun-Chieh Wu and Yi-Ting Chen). Three cancerous and one noncancerous tissue cores (diameter 2 mm) were longitudinally cut from each paraffin block and mounted with fine steel needles in new paraffin blocks to produce tissue microarrays.

Cancer tissue samples were cut into 4-μm-thick sections and deparaffinized in xylene as previously described [30]. Endogenous peroxidase activity was quenched with 3% (*v*/*v*) H_2_O_2_. To revive the antigens, the sections were boiled in 10 mM citrate buffer for 20 min. The tissues were incubated with 21 primary antibodies at room temperature for 30 min and then rinsed with phosphate-buffered saline (PBS) three times (Supplementary Table S1) according to the manufacturer’s protocol. The 21 target proteins included FEN1 (cytoplasmic staining, cy), PARP1 (nuclear staining, nu), FLNA (nu and cy), PIM1 (nu and cy), STK17A (nu and cy), CDH3 (nu and cy), SHC1 (nu and cy), P53 (nu), POLB (nu and cy), RAD54B (nu), SGK2 (cy), PhosphoMet (nu and cy), CNSK1E (cy), PLK1 (cy), CDK6 (nu and cy), Kras (cy), BRCA1 (nu), MSH2 (nu), EGFR (membranous and cytoplasmic staining), RB1 (nu and cy), and P16 (nu and cy), for 32 protein profiles with different staining locations. Next, the tissues were incubated at 25 °C for 30 min with secondary antibodies and a horseradish peroxidase/Fab polymer conjugate [EnVision™ detection systems peroxidase/DAB, rabbit/mouse (K5007 HRP; DaKo; Agilent Technologies, Inc., Santa Clara, CA, USA), and then thrice rinsed with PBS. Finally, the chromogen was developed using 3,3′-diaminobenzidine tetrahydrochloride as the substrate, counterstained with hematoxylin, and viewed under a microscope. Staining intensity was independently examined by two pathologists (Chun-Chieh Wu and Yi-Ting Chen).

The scoring criteria used were the same as those previously described [32]. Staining intensity was graded as negative (0), indeterminate (±), weakly positive (1+), moderately positive (2+), or strongly positive (3+). Negative (0) indicates no expression of the detected protein, indeterminate means that the staining is weak and its percentage cannot be accurately counted, weakly positive indicates <5% expression of the detected protein, moderately positive implies a focal expression in 5–20% of the cancer cells, and strongly positive indicates diffuse expression in >20% of the cancer cells. For the cancer tissue, staining intensity was compared with that of noncancerous oral mucosa, categorized as either overexpression or underexpression. Results of duplicate cores of each cancer tissue were combined to give a tumor score. When the two scores differed, the mean of the two scores was used as the overall tumor score. Cores were considered assessable if there was enough tumor tissue for evaluating the immunohistochemical staining. If one core was not assessable, the overall tumor score was the mean of the remaining assessable cores. Cores were regarded as not assessable in case of sampling error (<10% tumor cells in the core, for example only stroma) or absent core (<10% of the tissue was present in the core). For each protein, the staining results of each patient were visualized using a heatmap plot with normalized staining scores (Figure 1).

### 2.4. Ward’s Agglomerative Hierarchical Clustering

Overall, 32 protein staining expressions from 102 patients were normalized and converted into a 32 × 102 matrix. Agglomerative hierarchical clustering with Ward’ s method was used to cluster the protein staining expression matrix to build a hierarchy for included protein staining. Ward’s agglomerative hierarchical clustering algorithm divided the protein staining expression into *n* partitions according to their similarity. Silhouette analysis was used to estimate the optimal number of clusters for the input *n* × *m* matrix by estimating the average distance between clusters. The silhouette index $s_{i}$ measures the similarity between clusters and indicates whether the clustering configuration is appropriate. The protein staining hierarchical clustering was simply divided into three steps. We started with each object in an *n* × *m* matrix. Second, we used the merge cost formula shown in Equation (2) to ascertain the closest pair of clusters by merging the minimum merge cost objects. Third, the tree of cluster merges was returned and the second step was repeated until all objects were merged in the optimal number of clusters measured by the silhouette index. Thus, each cluster $C_{j}$ includes *k* number of hierarchy protein *P* with staining expression. The detail algorithm and description for the Ward’s agglomerative hierarchical clustering algorithm are described in Supplementary Materials.

### 2.5. OCPRS

The detail algorithm and description for OCPRS are described in Supplementary Materials. Hence, a risk stratification formula was derived to provide a quick and simple risk estimation using PLK1_cy, PhosphoMet_cy, and SGK2_cy staining results.

The agglomerative distance $D_{h}\;$for high-risk strata was computed as follows:

(1) $$D_{h}=||P_{\mathrm{PLK}1\_\mathrm{cy}}-{\bar{H}}_{\mathrm{PLK}1\_\mathrm{cy}}||+||P_{\mathrm{PhosphoMet}\_\mathrm{cy}}-{\bar{H}}_{\mathrm{PhosphoMet}\_\mathrm{cy}}||+||P_{\mathrm{SGK}2\_\mathrm{cy}}-{\bar{H}}_{\mathrm{SGK}2\_\mathrm{cy}}||$$

The mean of PLK1_cy, PhosphoMet_cy, and SGK2_cy in the high-risk cluster were 1.490, 0.962, and 0.981, respectively. Thus, $D_{h}$ was computed as follows:

(2) $$D_{h}=||P_{\mathrm{PLK}1\_\mathrm{cy}}-1.490||+||P_{\mathrm{PhosphoMet}\_\mathrm{cy}}-0.962||+||P_{\mathrm{SGK}2\_\mathrm{cy}}-0.981||$$

The agglomerative distance $D_{l}\;$for low-risk strata was computed as follows:

(3) $$D_{l}=||P_{\mathrm{PLK}1\_\mathrm{cy}}-{\bar{L}}_{\mathrm{PLK}1\_\mathrm{cy}}||+||P_{\mathrm{PhosphoMet}\_\mathrm{cy}}-{\bar{L}}_{\mathrm{PhosphoMet}\_\mathrm{cy}}||+||P_{\mathrm{SGK}2\_\mathrm{cy}}-{\bar{L}}_{\mathrm{SGK}2\_\mathrm{cy}}||$$

The mean of PLK1_cy, PhosphoMet_cy, and SGK2_cy in the low-risk cluster were 2.310, 1.840, and 1.590, respectively. Thus, $D_{l}$ was computed as follows.

(4) $$D_{l}=||P_{\mathrm{PLK}1\_\mathrm{cy}}-2.310||+||P_{\mathrm{PhosphoMet}\_\mathrm{cy}}-1.840||+||P_{\mathrm{SGK}2\_\mathrm{cy}}-1.590||$$

Lastly, each patient was dichotomized into high- and low-risk strata by comparing $D_{h}$ and $D_{l}$ using Equation (S10) (Supplementary Materials).

### 2.6. Statistical Analyses

The patient baseline characteristics are presented as frequency, percentage, or mean and standard deviation (SD). Survival outcomes, including PFS and OS, between high- and low-risk strata derived by OCPRS, were analyzed using the Kaplan–Meier method. Survival differences between high- and low-risk strata were tested using the log-rank test. A Cox proportional hazard regression model was used to identify the independent risks of baseline characteristics and OCPRS risk strata for survival outcomes. All statistical analyses were two-sided, and a *p*-value < 0.05 was considered statistically significant, performed using the computing environment R 3.5.3 (R Core Team, 2019).

## 3. Results

### 3.1. Clinicopathological Characteristics and Progression of Oral Cancer

Table 1 summarizes the clinicopathological characteristics of patients with oral cancer in our cohort. The mean age of patients with OSCC was 55.1 ± 10.4, and 94.1% were male. Overall, 37 patients demonstrated alcohol addiction (36.3%), 75 patients admitted betel nut use (73.5%), and 87 patients were tobacco users (85.3%). Most primary sites were of buccal origin (58.8%). According to the pathological grading system, grade 1 was observed in 48 patients, grade 2 in 52 patients, and grade 3 in 2 patients. A positive margin of the surgical specimen was observed in 6 patients, LVI in 10 patients, PNI in 13 patients, and ENE in 9 patients. The mean OSCC tumor size was 2.4 ± 1.5 cm. Seventy-six patients (74.5%) presented positive lymph node invasion. According to the 8th edition of the AJCC/UICC TNM staging system [33,34], pathological stages I and II were observed in 61 patients, with pathological stages III and IV observed in 41 patients. Finally, 26 patients died, and disease progression was documented in 36 patients during the follow-up period.

### 3.2. Hierarchical Clustering Analysis of the Optimal Combination of Protein Staining

**Figure 2:**
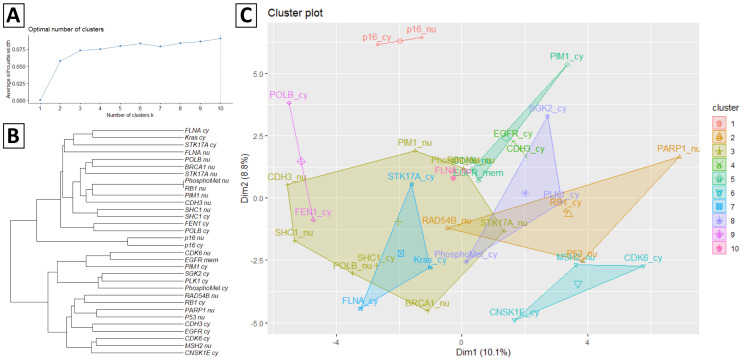
Ward’s agglomerative hierarchical results of the protein staining expression matrix. (**A**) The average silhouette width of each cluster and the dash line indicates the optimal number of clusters according to the silhouette index. (**B**) Dendrogram of hierarchical clustering results of 32 protein staining. (**C**) Cluster plot of protein staining coloring according to the assigned cluster.

Figure 2 presents Ward’s agglomerative hierarchical clustering results according to the protein staining expression matrix illustrated in Figure 1. Figure 2A illustrates the average silhouette width of each cluster using a line plot, and the dashed line indicates the optimal number of clusters is ten according to the silhouette index. Figure 2B presents the dendrogram of hierarchical clustering results of 32 protein stainings. Figure 2C visualizes the protein staining in a scatter plot with each protein staining colored according to its assigned cluster.

### 3.3. Hierarchical Clustering Results of Protein Staining

During the follow-up period, 36 subjects experienced disease progression, and 26 fatalities were documented. Table 2 summarizes the distribution of death and progressed subjects according to the agglomerative distance dichotomous results. Two of ten protein staining clusters derived from the hierarchical clustering analysis showed significant survival differences in PFS or OS. Within the 8th protein staining cluster (including PLK1_cy, PhosphoMet_cy, and SGK2_cy), 53 and 49 subjects were dichotomized into high-risk and low-risk strata, respectively. The high-risk strata of the 8th cluster identified 75.0% (25 of 36) of progressed subjects and 76.9% (20 of 26) of dead subjects. The log-rank test results demonstrated that the high-risk strata demonstrated a significant survival difference when compared to low-risk strata in PFS (*p* = 0.012). Although the 5th protein staining cluster (including EGFR_mem, CDK6_nu, and PIM1_cy) showed significant survival differences in OS between strata (*p* = 0.015), identifying 100% (36 of 36) progressed subjects and 96.2% (25 of 26) dead subjects in high-risk strata, the results were attributed to the extremely imbalanced dichotomous results between high-risk (101 subjects) and low-risk (1 subject) strata. Hence, the 5th protein staining cluster was excluded in further analysis. The results demonstrated that protein staining, including PLK1_cy, PhosphoMet_cy, and SGK2_cy, could significantly predict oral cancer progression.

**Figure 3:**
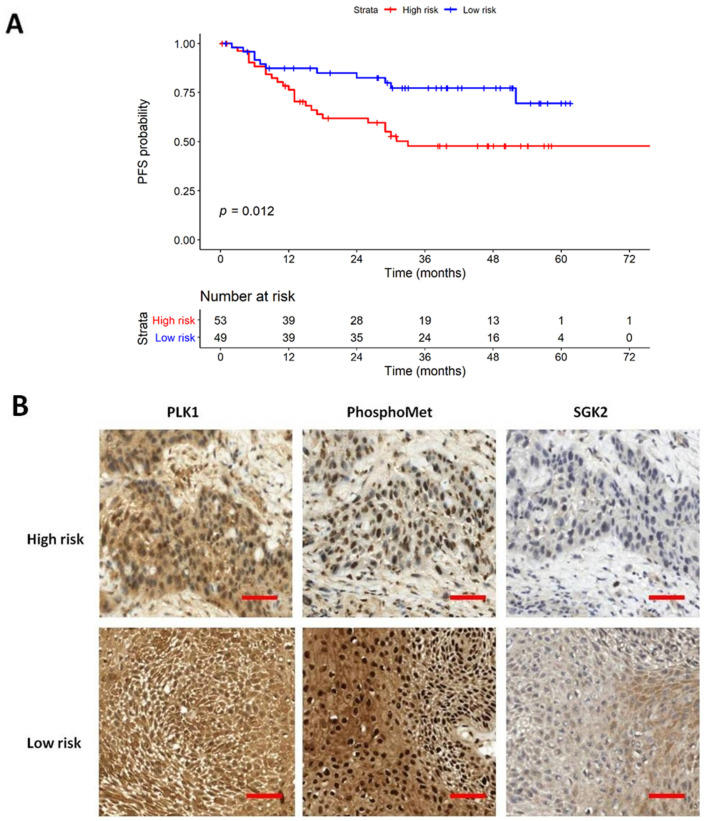
Kaplan–Meier plot of progression-free survival (PFS). (**A**) PFS results stratified by 8th protein staining cluster. (**B**) IHC staining of 8th protein staining cluster of high-risk and low-risk patients, respectively. The scale bar showed 200 μm in distance (magnification 400×).

**Figure 4:**
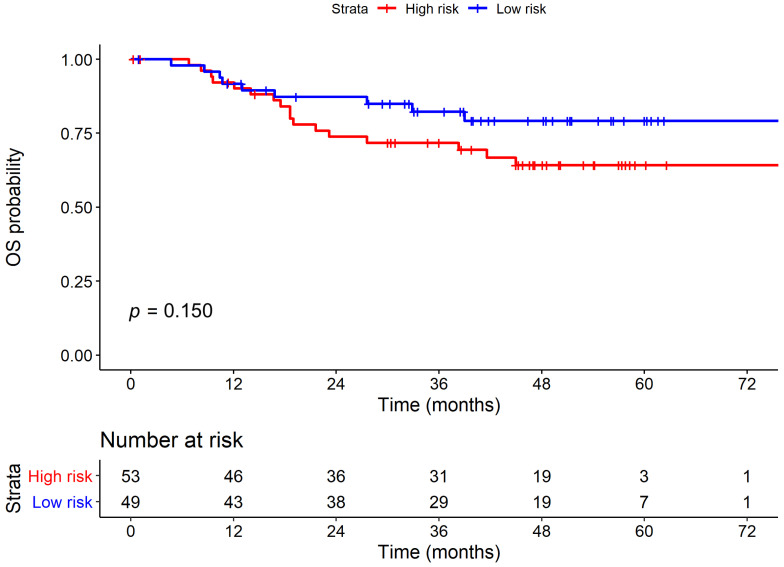
Kaplan–Meier plot of overall survival (OS) stratified by 8th protein staining cluster.

Figure 3A compares the survival curves between high-risk and low-risk strata derived from the 8th protein staining cluster. The high-risk strata showed a significantly poor PFS when compared to low-risk strata. Figure 3B and Supplementary Figure S1 represent typical features of IHC staining and associated H&E images in high-risk and low-risk patients. However, the 8th protein staining cluster demonstrated no significant results in OS analysis, as shown in Figure 4. However, there are no differences in baseline characteristics between these two groups except for disease progression (47.2% vs. 22.4%, *p* = 0.016), which is shown in Supplementary Table S2.

The PFS and OS results using the Cox proportional hazard regression analysis are summarized in Table 3 and Table 4, respectively. In the survival analysis, the 8th protein staining cluster was included and analyzed using the common survival predictors. In PFS, the high-risk subjects demonstrated a significantly increased risk for disease progression when compared with low-risk subjects in both univariate (hazard ratio (HR) = 2.41, 95% confidence interval (CI) = 1.19–4.91, *p* = 0.015) and multivariate (HR = 2.33, 95% CI = 1.14–4.75, *p* = 0.020) analyses. In the multivariate model, patients with ENE (HR = 2.66, 95% CI = 1.02–6.95, *p* = 0.045) demonstrated a significantly increased disease progression risk. In addition, we provided Supplementary Table S3 for comparison of the prediction ability of different protein location on the overall mortality and disease progression. In our analysis, different localizations of 11 proteins had little impact on mortality and disease progression.

In OS, no significant difference was observed in the risk of death between high-risk and low-risk subjects in the univariate (HR = 1.79, 95% CI = 0.80–4.02, *p* = 0.157) analyses. However, OS was highly associated with common clinical factors, including grade and ENE. Patients with higher histological grade demonstrated a significantly increased mortality risk when compared with the subjects presenting lower histological grade in both univariate (hazard ratio [HR] = 3.65, 95% confidence interval (CI) = 1.46–9.10, *p* = 0.006) and multivariate (HR =3.05, 95% CI = 1.17–7.90, *p* = 0.022) OS analyses. Patients with ENE showed a significantly increased mortality risk when compared with those without ENE in both univariate (hazard ratio (HR) = 6.94, 95% confidence interval [CI] = 2.71–17.82, *p* < 0.001) and multivariate (HR = 3.46, 95% CI = 1.05–11.41, *p* = 0.042) OS analyses. Collectively, stratification via the 8th protein staining cluster (including PLK1_cy, PhosphoMet_cy, SGK2_cy) demonstrated a novel predictor of disease progression in oral cancer.

## 4. Discussion

Oral cancer is a multifactorial malignancy. Several studies have evaluated demographic, epidemiological, histopathological, and molecular prognostic factors that could impact disease outcomes [2]. Previously, some researchers have analyzed the correlation between different factors and prognosis; however, none can individually influence the prognosis of patients with oral cancer. Determining the outcomes and prognosis in patients with oral cancer should incorporate diverse aspects and statistical methods. The hierarchical agglomerative clustering algorithm could effectively recognize the interaction between high-dimensional protein staining matrices by considering similarities in protein clusters. A corresponding risk module, OCPRS risk estimation modules, is derived according to the agglomerative clustering results, enabling the generalization of current study IHC findings in clinical settings. Furthermore, the OCPRS modules could be applied to survival analysis, including the Cox model, to investigate the simultaneous impact of baseline clinical characteristics and OCPRS risk on the survival outcome.

In our study, we collected OSCC demographic data, staging, imaging, surgical interventions, pathological interpretations, survivals, and outcomes concerning 163 patients, and analyzed the profiles of 102 patients. All patients underwent surgery, performed according to national guidelines in centralized settings, with adequate specimens acquired. No singular factor interfered with the progression and survival outcomes. This is favorable, suggesting that the prognosis of patients with OSCC should not consider solitary factors or single markers. Next, we incorporated all clinicopathological features and IHC staining results and utilized the hierarchical clustering analysis to determine optimal combinations of protein staining. We stratified patients into high-risk and low-risk groups according to the 8th protein staining cluster (including PLK1_cy, PhosphoMet_cy, and SGK2_cy). Finally, we developed a unique and novel approach to adopt the prognostic usefulness of a scoring system, OCPRS, for the diagnosis of patients with oral cancer. The OCPRS was generated based on a hierarchical agglomerative algorithm, sensitive to background noise that allows a decrease in type 1 errors (false positive). Consistent with previous studies, we demonstrated that the agglomerative hierarchical clustering algorithm is advantageous for handling high-dimensional data with uncertain interactions between factors [35,36,37]. OCPRS could recognize the interaction between factors by considering the similarities within a protein cluster.

Furthermore, we investigated and predicted disease progression using the Cox proportional hazards regression analysis, controlled for the influence of clinicopathological features, indicating that patients with high-risk cancer were 2.33 times more likely to experience cancer progression than those with low-risk cancer (95% CI for the hazard ratio = 1.14–4.75; *p* = 0.020). However, similar results were not observed in OS (HR = 1.79, 95% CI = 0.80–4.02, *p* = 0.157) in univariate analysis. Patients with ENE demonstrated an increased risk of progression in both univariate (*p* = 0.030) and multivariate models (*p* = 0.045). Patients with higher histological grade and ENE demonstrated an increased mortality risk in both the univariate (*p* = 0.006 and *p* < 0.001) and multivariate models (*p* = 0.022 and *p* = 0.042, respectively). Patients with higher histological grade presented a higher risk of progression in the univariate model (*p* = 0.043), with no significant effects in the multivariate model (*p* = 0.079). Patients with PNI, larger tumor size, lymph node invasion, and pathological stage demonstrated an increased mortality risk in the univariate model, with no significant effects in the multivariate model. These results suggest that there exist interactions that interfere with these clinicopathological factors [38,39].

The EGFR and CDK6 were found significantly associated with the overall survival in 5th protein staining cluster (including EGFR_mem, CDK6_nu, and PIM1_cy), which could identify 100% (36 of 36) progressed subjects and 96.2% (25 of 26) dead subjects in high-risk strata. However, the results were attributed to the extremely imbalanced dichotomous results between high-risk (101 subjects) and low-risk (1 subject) strata. The results indicate the EGFR and CDK6 might obtain a good sensitivity to detect high-risk patients, but the low specificity to survival outcome estimation leads to the omission of EGFR and CDK6 in current study. Thence, further research to further investigate the interaction between EGFR, CDK6, and others factors using machine learning approaches such as evolutionary and optimization algorithm is necessary to identify the high-risk subjects more accurately.

Besides, our study raised an interesting issue of localization of IHC staining. In previous studies of ovarian cancer, different localizations of thyroid hormone receptors and αvβ3 integrin demonstrated different nuclear and non-nuclear signaling pathway for cancerogenesis [40,41]. The translocation of transmembrane proteins from the cell membrane to the nucleus may be another crucial mechanism [42]. In oral and oropharyngeal cancer, different membrane and cytoplasmic CD44 expressions also determined distinct clinical outcomes [43]. In head and neck cancer, translocalization of ING3 affected tumorigenesis and cancer progression [44]. However, overall mortality and disease progression appeared unaffected by different localizations of 11 proteins in our study.

## 5. Conclusions

Here, we developed a new statistical approach using hierarchical clustering. By combining predictive IHC biomarkers, we newly defined OCPRS to predict PFS outcomes for oral cancer. OCPRS is clinically available and easily measurable for the staining of surgical specimens. High-risk OCPRS with evaluation of cytoplasmic PLK1, PhosphoMet, and SGK2 staining is useful for the stratification of clinical outcomes. Therefore, OCPRS may be used as a promising biomarker for predicting progression outcomes and stratifying risk groups for oral cancer.

## Figures and Tables

**Table 1:** Baseline characteristics.

Characteristics	*n* (%)
Cases	102
Age, mean ± SD	55.1 ± 10.4
Sex	
Female	6 (5.9%)
Male	96 (94.1%)
Alcohol	37 (36.3%)
Betel	75 (73.5%)
Cigarette	87 (85.3%)
Site	
Non-buccal	42 (41.2%)
Buccal	60 (58.8%)
Grade	
1	48 (47.1%)
2	52 (51%)
3	2 (2%)
LVI	10 (9.8%)
PNI	13 (12.7%)
Margin not free	6 (5.9%)
ENE	9 (8.8%)
Tumor size (cm), mean ± SD	2.4 ± 1.5
Lymph node invasion	
Positive	76 (74.5%)
Negative	26 (25.5%)
Pathological stage	
I–II	61 (59.8%)
III–IV	41 (40.2%)
Death	26 (25.5%)
Progressed	36 (35.3%)

**Table 2:** Distribution of died and progressed subjects according to the agglomerative distance dichotomous results.

No	Cluster	High-Risk Strata	n ^a^ (%)	n ^b^ (%)	Low-Risk Strata	n ^a^ (%)	n ^b^ (%)	*p* ^a^	*p* ^b^
1	p16_cy, p16_nu	88	29 (80.6%)	23 (88.5%)	14	7 (19.4%)	3 (11.5%)	0.182	0.764
2	RB1_cy, RAD54B_nu, P53_nu, PARP1_nu	49	20 (55.6%)	15 (57.7%)	53	16 (44.4%)	11 (42.3%)	0.341	0.350
3	RB1_nu, BRCA1_nu, PhosphoMet_nu, POLB_nu, SHC1_cy, SHC1_nu, CDH3_nu, STK17A_nu, PIM1_nu	64	25 (69.4%)	19 (73.1%)	38	11 (30.6%)	7 (26.9%)	0.427	0.283
4	EGFR_cy, CDH3_cy	91	34 (94.4%)	25 (96.2%)	11	2 (5.6%)	1 (3.8%)	0.151	0.212
5	EGFR_mem, CDK6_nu, PIM1_cy	101	36 (100%)	25 (96.2%)	1	0 (0%)	1 (3.8%)	0.619	0.015 *
6	MSH2_nu, CDK6_cy, CNSK1E_cy	68	22 (61.1%)	16 (61.5%)	34	14 (38.9%)	10 (38.5%)	0.161	0.354
7	Kras_cy, STK17A_cy, FLNA_cy	85	27 (75.0%)	20 (76.9%)	17	9 (25.0%)	6 (23.1%)	0.087	0.354
8	PLK1_cy, PhosphoMet_cy, SGK2_cy	53	25 (69.4%)	17 (65.4%)	49	11 (30.6%)	9 (34.6%)	0.012 *	0.152
9	POLB_cy, FEN1_cy	66	25 (69.4%)	18 (69.2%)	36	11 (30.6%)	8 (30.8%)	0.279	0.379
10	FLNA_nu	64	22 (61.1%)	18 (69.2%)	38	14 (38.9%)	8 (30.8%)	0.649	0.644

n ^a^ and n ^b^ indicate the progressed and died numbers of subjects within strata, respectively. *p* ^a^ and *p* ^b^ indicate the log-rank test *p*-value of progression-free and overall free survival, respectively. * Statistically significant (*p* < 0.05).

**Table 3:** Cox proportional hazard regression analysis of progression-free survival.

Predictors	Comparison	Univariate		Multivariate	
		HR (95%CI)	*p*	HR (95%CI)	*p*
8th protein staining cluster	High vs. low-risk	2.41 (1.19–4.91)	0.015 *	2.33 (1.14–4.75)	0.020 *
Age	Years	1.01 (0.98–1.04)	0.500	-	
Sex	Male vs. female	1.17 (0.28–4.88)	0.828	-	
Alcohol	Yes vs. no	1.01 (0.51–2.00)	0.971	-	
Betel	Yes vs. no	0.78 (0.38–1.58)	0.487	-	
Cigarrate	Yes vs. no	0.86 (0.36–2.07)	0.735	-	
Site	Buccal vs. non-buccal	1.44 (0.73–2.85)	0.290	-	
Grade	Grade 2–3 vs. 1	2.03 (1.02–4.01)	0.043 *	1.85 (0.93–3.67)	0.079
LVI	Yes vs. no	1.92 (0.75–4.95)	0.176	-	
PNI	Yes vs. no	1.99 (0.87–4.55)	0.104	-	
Margin	Not free vs. free	1.59 (0.49–5.20)	0.443	-	
ECS	Yes vs. no	2.87 (1.11–7.46)	0.030 *	2.66 (1.02–6.95)	0.045 *
Tumor size	cm	1.02 (0.81–1.28)	0.863	-	
Lymph node invasion	Positive vs. negative	1.30 (0.63–2.70)	0.479	-	
Pathological stage	III–IV vs. I–II	1.71 (0.89–3.30)	0.109	-	

Predictors with *p* < 0.10 in univariate analysis is included in multivariate analysis. * Statistically significant (*p* < 0.05).

**Table 4:** Cox proportional hazard regression analysis of overall survival.

Predictors	Comparison	Univariate		Multivariate	
		HR (95%CI)	*p*	HR (95%CI)	*p*
8th protein staining cluster	High vs. low-risk	1.79 (0.80–4.02)	0.157	-	
Age	Years	1.00 (0.96–1.04)	0.868	-	
Sex	Male vs. female	1.61 (0.22–11.91)	0.640	-	
Alcohol	Yes vs. no	1.82 (0.73–4.53)	0.200	-	
Betel	Yes vs. no	0.52 (0.23–1.14)	0.100	-	
Cigarrate	Yes vs. no	2.08 (0.49–8.81)	0.319	-	
Site	Buccal vs. non-buccal	1.09 (0.50–2.38)	0.824	-	
Grade	Grade 2–3 vs. 1	3.65 (1.46–9.10)	0.006 *	3.05 (1.17–7.90)	0.022 *
LVI	Yes vs. no	2.19 (0.75–6.38)	0.151		
PNI	Yes vs. no	3.84 (1.66–8.89)	0.002 *	2.31 (0.82–6.47)	0.111
Margin	Not free vs. free	2.09 (0.63–6.96)	0.232		
ECS	Yes vs. no	6.94 (2.71–17.82)	<0.001 *	3.46 (1.05–11.41)	0.042 *
Tumor size	cm	1.38 (1.13–1.70)	0.002 *	1.16 (0.86–1.55)	0.334
Lymph node invasion	Positive vs. negative	3.14 (1.45–6.81)	0.004 *	0.87 (0.26–2.96)	0.829
Pathological stage	III–IV vs. I–II	3.13 (1.39–7.02)	0.006 *	1.45 (0.42–4.98)	0.557

Predictors with *p* < 0.10 in univariate analysis is included in multivariate analysis. * Statistically significant (*p* < 0.05).

## Data Availability

The data presented in this study are available on request from the corresponding author.

## Supplementary Materials

The following are available online at https://www.mdpi.com/article/10.3390/diagnostics11060925/s1, Figure S1. IHC staining (magnification 400×) of 8th protein staining cluster and associated H&E images (magnification 200×) of high-risk and low-risk patients, Table S1. The antibodies and retrieval buffers for each protein, Table S2. Baseline characteristics according to identified protein cluster, Table S3. Comparison of the prediction ability of different protein location on overall mortality and disease-progressed.
