# Supplementary material for: Application of the Interaction between Tissue Immunohistochemistry Staining and Clinicopathological Factors for Evaluating the Risk of Oral Cancer Progression by Hierarchical Clustering Analysis: A Case-Control Study in a Taiwanese Population
Source: Diagnostics (Basel). 2021 May 21;11(6):925. doi: 10.3390/diagnostics11060925 (PMC8224004; doi:10.3390/diagnostics11060925)
Supplement: Supplementary file 1 [file diagnostics-11-00925-s001.zip › diagnostics-1218298-SI.pdf]

Ward's agglomerative hierarchical clustering

Overall, 32 protein staining expressions from 102 patients were normalized and converted into a 32×102 matrix. Agglomerative hierarchical clustering with Ward's method was used to cluster the protein staining expression matrix to build a hierarchy for included protein staining. Ward's agglomerative hierarchical clustering algorithm divided the protein staining expression into *n* partitions according to their similarity. Ward's agglomerative hierarchical clustering equation is shown in equations 1 and 2.

$$\Delta(A,B) = \sum_{i\in A\cup B} \|\vec{x} - \vec{m}_{A\cup B}\|^2 - \sum_{i\in A} \|\vec{x} - \vec{m}_A\|^2 - \sum_{i\in B} \|\vec{x} - \vec{m}_B\|^2 \quad (1)$$

$$= \frac{n_A n_B}{n_A + n_B} \|\vec{m}_A - \vec{m}_B\|^2 \quad (2)$$

where A and B indicate two different clusters, $\vec{m}_A$ is the center of cluster A, and n_A is the number of objects within cluster A. Δ is the merging cost of combining clusters A and B.

Silhouette analysis was used to estimate the optimal number of clusters for the input $n \times m$ matrix by estimating the average distance between clusters. The silhouette index s_i measures the similarity between clusters and indicates whether the clustering configuration is appropriate.

$$b(i) = \min_{c\neq A} c(i,C) \quad (3)$$

where *i* is an object belonging to cluster *A*, *C* is a cluster not containing *i*, and *c*(*i*, *C*) is the average dissimilarity between *i* and all objects in *C*. Hence, the silhouette index $s(i)$ is calculated as follows:

$$s(i) = \frac{b(i) - a(i)}{\max\{a(i), b(i)\}} \quad (4)$$

where *a*(*i*) is defined as the average dissimilarity between *i* and all other objects in *A.*

The protein staining hierarchical clustering was simply divided into three steps. We started with each object in a $n \times m$ matrix. Second, we used the merge cost formula shown in equation 2 to ascertain the closest pair of clusters by merging the minimum merge cost objects. Third, the tree of cluster merges was returned and the second step was repeated until all objects were merged in the optimal number of clusters measured by the silhouette index in equation 4. Thus, each cluster C_j will include *k* number of hierarchy protein *P* with staining expression.

$$C_j = \{P_1, \ldots, P_k\} \quad (5)$$

OCPRS

Subsequently, patients were dichotomized into two strata by each cluster C_j using the agglomerative distance, as shown in equation 6.

$$D_k = \sum_{P \in C_j} \|P_k - M_k\| \tag{6}$$

where M_k is the mean vector of C_j strata and *P* is the *k* object in the vector of the protein staining matrix.

The proportion of disease progression subjects was computed and compared to determine the high- and low-risk strata. Thus, the mean vector of C_j in high- and low-risk strata was computed as equation 7**.**

$$M_k = \begin{cases} \bar{H} = \dfrac{\sum x_h}{n_h} \\ \bar{L} = \dfrac{\sum x_l}{n_l} \end{cases} \tag{7}$$

where $\bar{H}$ is the protein staining mean score of high-risk strata, and $\bar{L}$ is the protein staining mean score of low-risk strata.

Consequently, the agglomerative distance D_k for high- and low-risk strata was calculated using equations 8 and 9.

$$D_h = \sum_{k \in C_j} \|P_k - \bar{H}_k\| \tag{8}$$

where D_h is the agglomerative distance from the high-risk derived by the selected protein staining score combination in C_j. *P* is the *k* object in the vector of protein staining matrix, and $\bar{H}_k$ is the protein staining mean score of high-risk strata in each of the proteins included in C_j.

$$D_l = \sum_{k \in C_j} \|P_k - \bar{L}_k\| \tag{9}$$

where D_l is the agglomerative distance from the low-risk derived by the selected protein staining score combination in C_j. *P* is the *k* object in the vector of protein staining matrix, and $\bar{L}_k$ is the protein staining mean score of low-risk strata in each of the proteins included in C_j.

Hence, a risk stratification formula was derived to provide a rapid and convenient risk estimation using the protein staining expression in the cluster C_j. Each patient was dichotomized into high- and low-risk strata by comparing D_h and, D_l as shown in equation 10.

$$Risk\ stratification = \begin{cases} High\ risk, if\ D_h < D_l \\ Low\ risk, if\ D_l < D_h \end{cases} \tag{10}$$

The results demonstrated that protein staining, including PLK1_cy, PhosphoMet_cy, and SGK2_cy, could significantly predict oral cancer progression. Hence, a risk stratification formula was derived to provide a quick and simple risk estimation using PLK1_cy, PhosphoMet_cy, and SGK2_cy staining results.

The agglomerative distance D_h for high-risk strata was computed as follows:

$$D_h = \left\|P_{\mathrm{PLK1\_cy}} - \bar{H}_{\mathrm{PLK1\_cy}}\right\| + \left\|P_{\mathrm{PhosphoMet\_cy}} - \bar{H}_{\mathrm{PhosphoMet\_cy}}\right\| + \left\|P_{\mathrm{SGK2\_cy}} - \bar{H}_{\mathrm{SGK2\_cy}}\right\| \tag{11}$$

The mean of PLK1_cy, PhosphoMet_cy, and SGK2_cy in the high-risk cluster were 1.490, 0.962, and 0.981, respectively. Thus, D_h was computed as follows:

$$D_h = \left\|P_{\mathrm{PLK1\_cy}} - 1.490\right\| + \left\|P_{\mathrm{PhosphoMet\_cy}} - 0.962\right\| + \left\|P_{\mathrm{SGK2\_cy}} - 0.981\right\| \tag{12}$$

The agglomerative distance D_l for low-risk strata was computed as follows:

$$D_l = \left\|P_{\mathrm{PLK1\_cy}} - \bar{L}_{\mathrm{PLK1\_cy}}\right\| + \left\|P_{\mathrm{PhosphoMet\_cy}} - \bar{L}_{\mathrm{PhosphoMet\_cy}}\right\| + \left\|P_{\mathrm{SGK2\_cy}} - \bar{L}_{\mathrm{SGK2\_cy}}\right\| \tag{13}$$

The mean of PLK1_cy, PhosphoMet_cy, and SGK2_cy in the low-risk cluster were 2.310, 1.840, and 1.590, respectively. Thus, D_l was computed as follows.

$$D_l = \left\|P_{\mathrm{PLK1\_cy}} - 2.310\right\| + \left\|P_{\mathrm{PhosphoMet\_cy}} - 1.840\right\| + \left\|P_{\mathrm{SGK2\_cy}} - 1.590\right\| \tag{14}$$

Lastly, each patient was dichotomized into high- and low-risk strata by comparing D_h and D_l using equation 10.

Supplementary Figure S1. IHC staining (magnification 400×) of 8th protein staining cluster and associated H&E images (magnification 200×) of high-risk and low-risk patients.

High risk

(a) PLK1 IHC staining (magnification 400×)

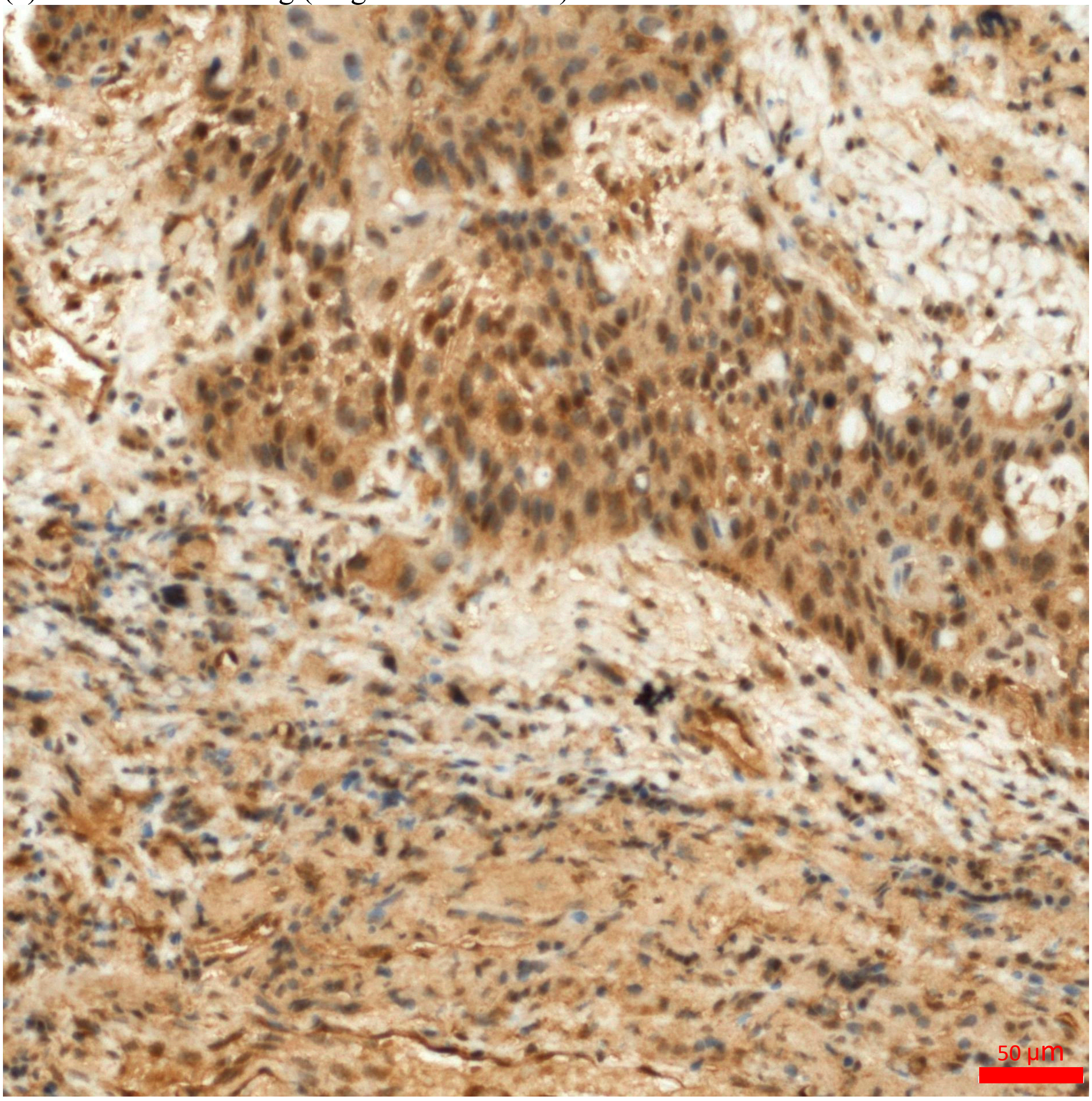

(b) phosphoMet IHC staining (magnification 400×)

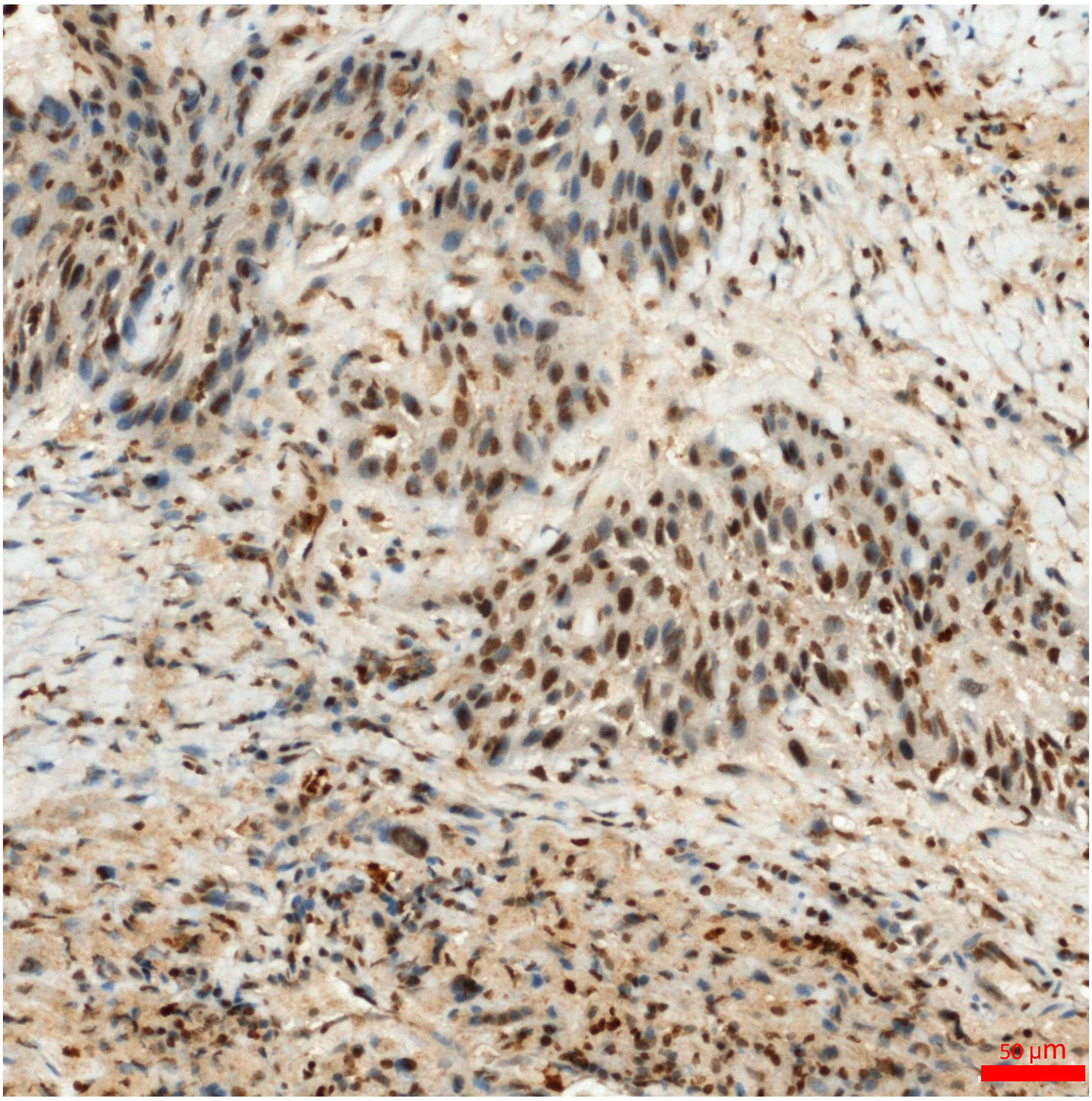

(c) SGK2 IHC staining (magnification 400×)

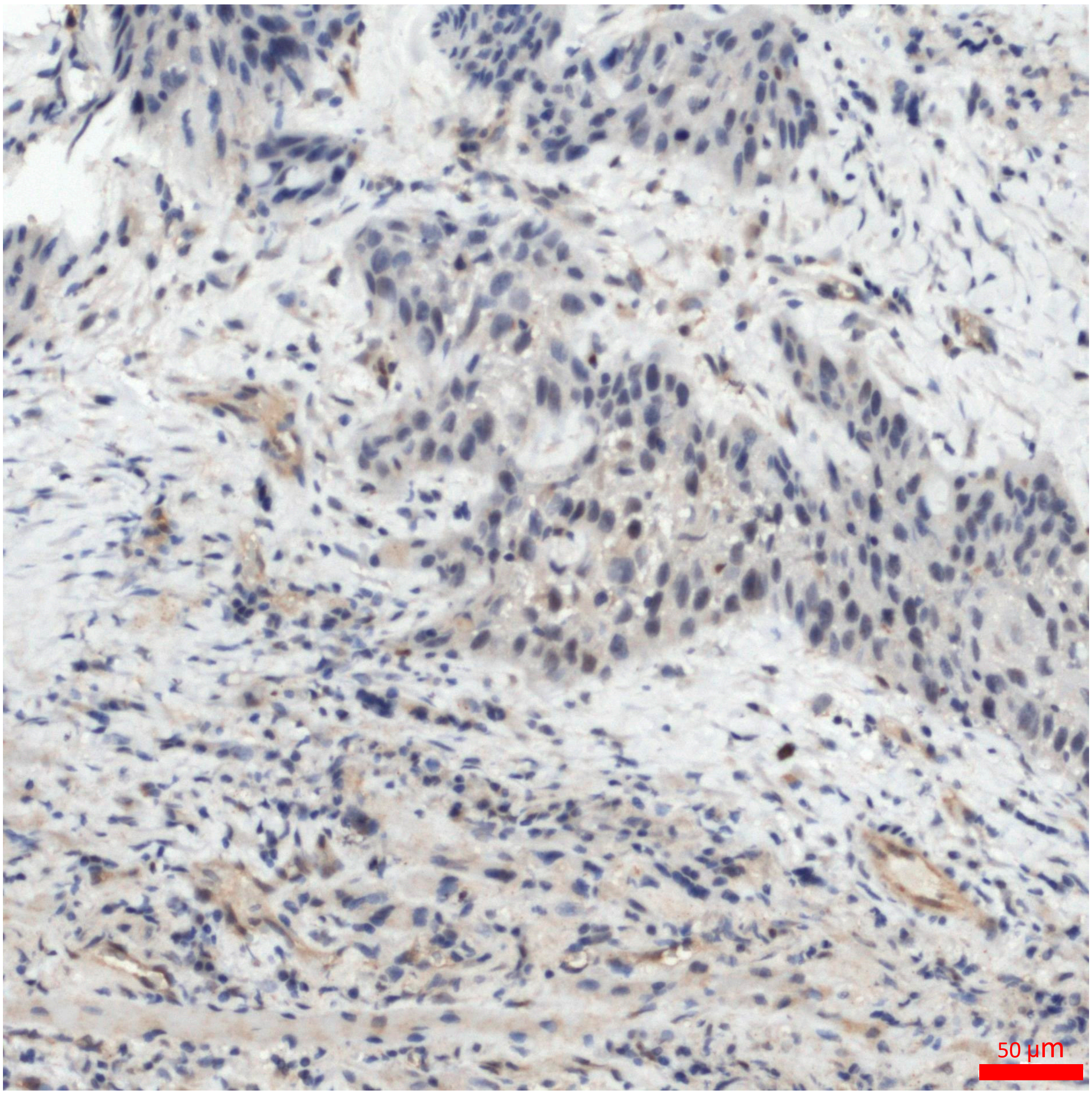

(d) Associated H&E images (magnification 200×)

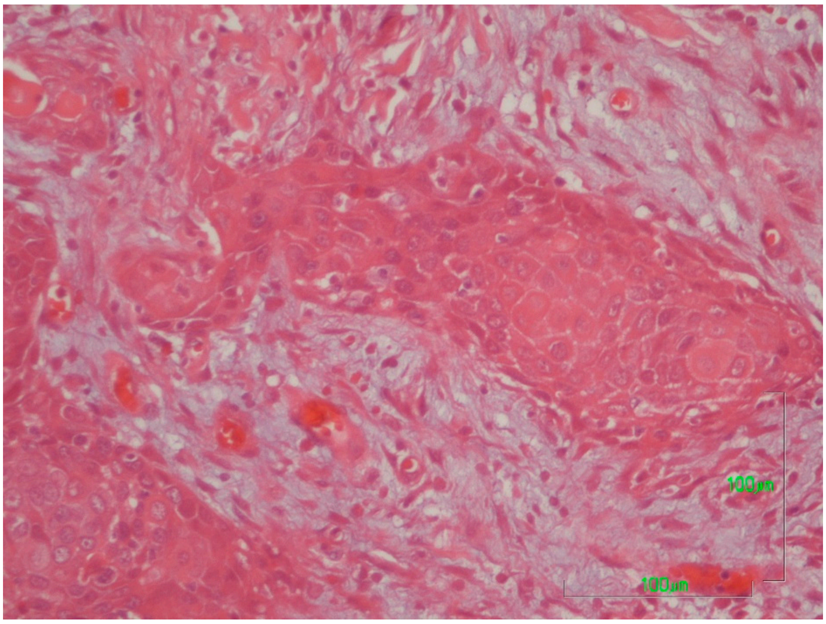

Low risk

(e) PLK1 IHC staining (magnification 400×)

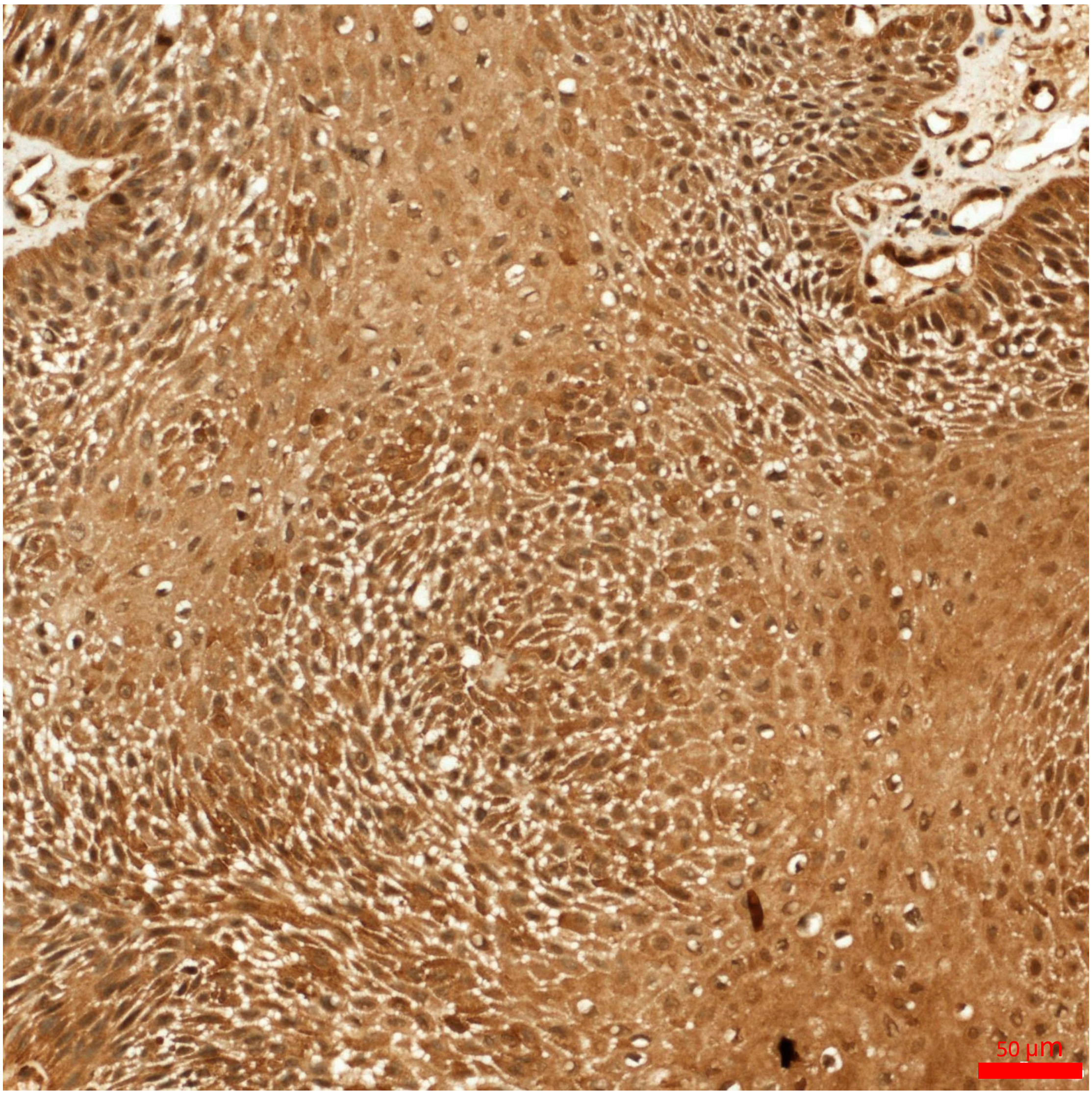

(f) phosphoMet IHC staining (magnification 400×)

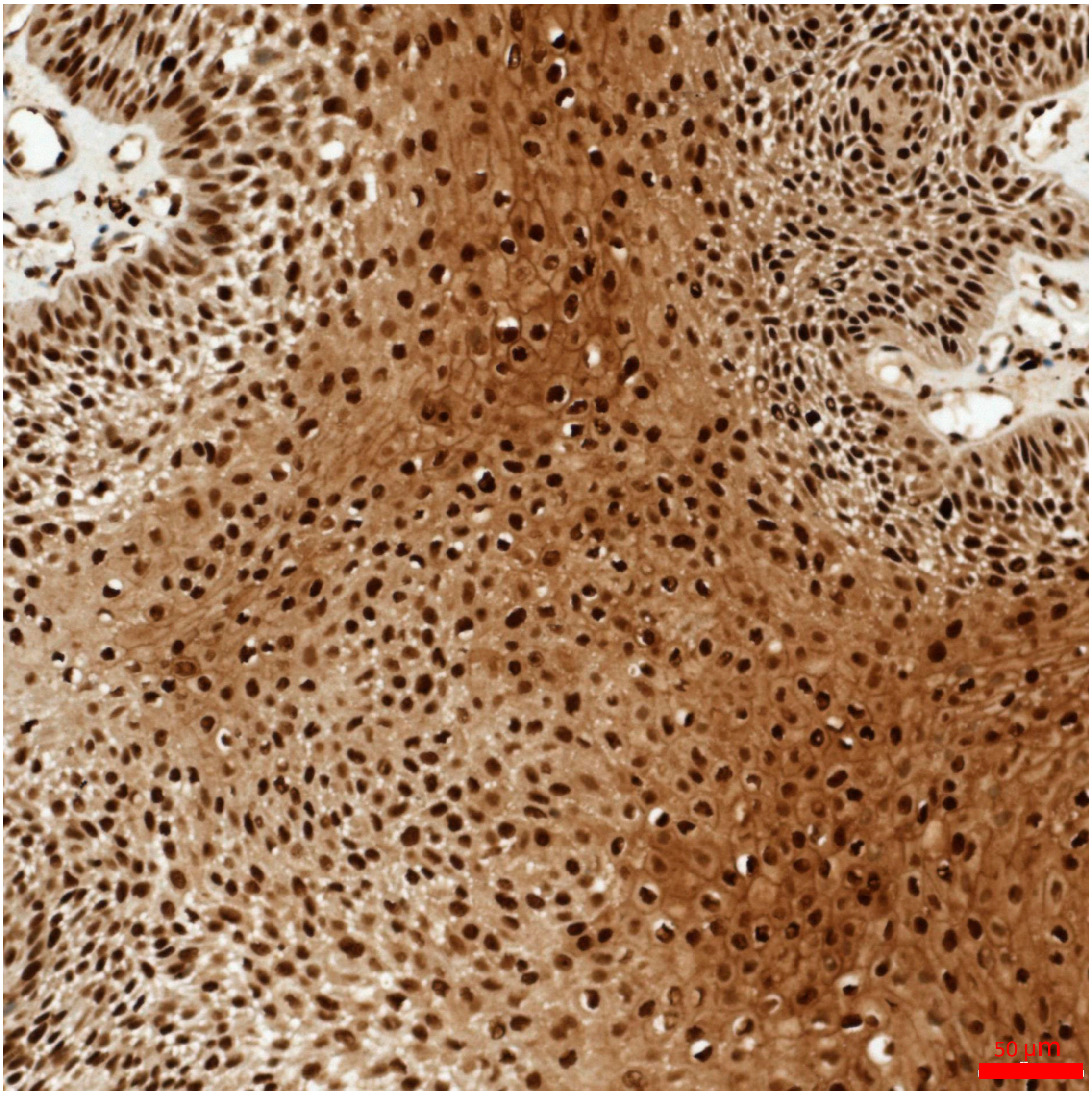

(g) SGK2 IHC staining (magnification 400×)

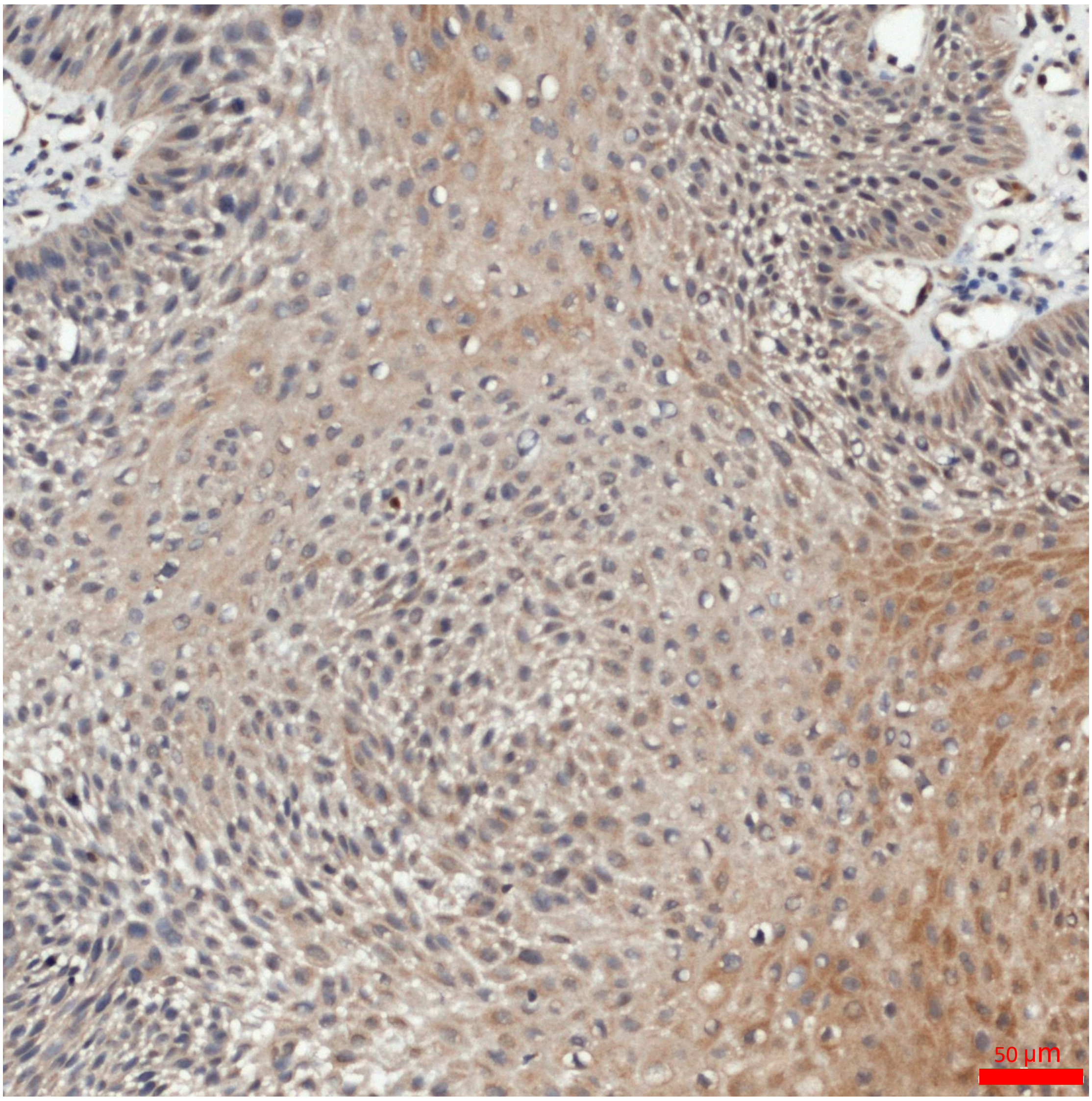

(h) Associated H&E images (magnification 200×)

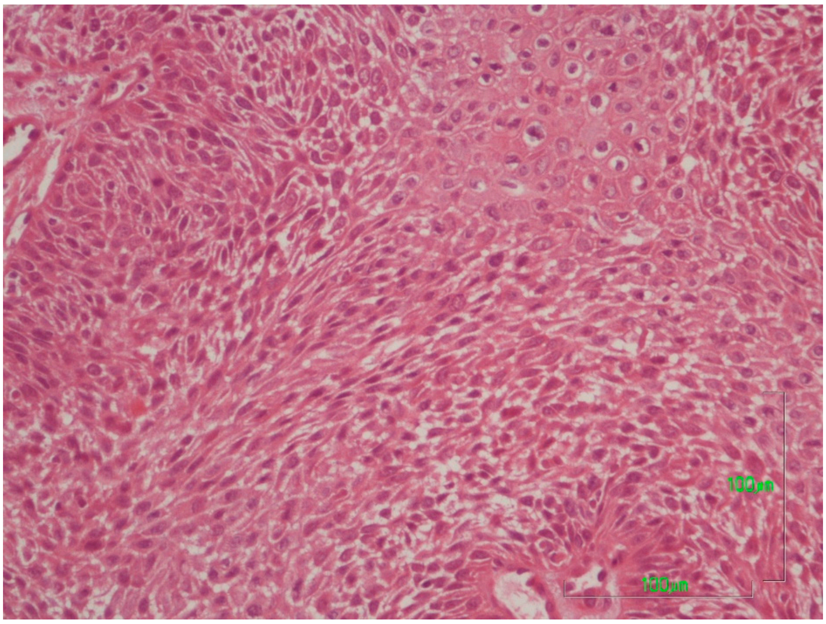

Supplementary Table S1. The antibodies and retrieval buffers for each protein.

Protein Name	Clonality	Source	Catalogue number	Dilution	Retrieval buffer
BRCA1	Mouse monoclonal	Zeta Corporation	Z2237	1:100	Tris-EDTA buffer
CDH3	Rabbit polyclonal	Abgent	AP1499B	1:50	Tris-EDTA buffer
CDK6	Rabbit monoclonal	Abcam Ltd	ab124821	1:100	Tris-EDTA buffer
CSNK1E	Rabbit polyclonal	Abgent	AP7403a	1:50	Tris-EDTA buffer
EGFR	Rabbit monoclonal	Zeta Corporation	Z2037	1:50	Tris-EDTA buffer
FEN1	Rabbit polyclonal	Abcam Ltd	ab70815	1:1000	Tris-EDTA buffer
FLNA	Rabbit polyclonal	Abgent	AP7770a	1:50	Tris-EDTA buffer
KRAS	Rabbit polyclonal	Abcam Ltd	ab216890	1:200	Citrate buffer
MET	Rabbit polyclonal	Abgent	AP3167a	1:50	Citrate buffer
MSH2	Mouse monoclonal	Zeta Corporation	Z2129	1:100	Tris-EDTA buffer
P16	Mouse monoclonal	BD biosciences	550834	1:100	Tris-EDTA buffer
PARP1	Rabbit monoclonal	Abcam Ltd	Ab191217	1:500	Tris-EDTA buffer
PIM1	Rabbit polyclonal	Abgent	AP7932d	1:50	Tris-EDTA buffer
PLK1	Rabbit polyclonal	Abgent	AP7937a	1:100	Citrate buffer
POLB	Rabbit polyclonal	Abgent	AP50642	1:100	Tris-EDTA buffer
RAD54B	Rabbit polyclonal	Genetex	GTX103291	1:500	Tris-EDTA buffer
RB1	Mouse monoclonal	Leica Biosystems	NCL-L-RB-358	1:50	Tris-EDTA buffer
SGK2	Rabbit polyclonal	Abgent	AP7947b	1:100	Citrate buffer
SHC1	Rabbit polyclonal	Abgent	AP50024	1:100	Citrate buffer
STK17A	Rabbit polyclonal	Abcam Ltd	ab97530	1:100	Citrate buffer
TP53	Mouse monoclonal	Leica Biosystems	NCL-L-p53-DO7	1:200	Citrate buffer

Supplementary Table S2. Baseline characteristics according to identified protein cluster.

Characteristics	High-risk	Low-risk	*P*
Cases	53	49	
Age, mean ± SD	54.5 ± 11.2	55.8 ± 9.5	0.514
Sex			0.102
Female	1 (1.9%)	5 (10.2%)	
Male	52 (98.1%)	44 (89.8%)	
Alcohol	34 (64.2%)	31 (63.3%)	1.000
Betel	40 (75.5%)	35 (71.4%)	0.812
Cigarette	48 (90.6%)	39 (79.6%)	0.199
Site			0.500
Non-buccal	24 (45.3%)	18 (36.7%)	
Buccal	29 (54.7%)	31 (63.3%)	
Grade			0.567
1	23 (43.4%)	25 (51.0%)	
2-3	30 (56.6%)	24 (49.0%)	
LVI	7 (13.2%)	3 (6.1%)	0.323
PNI	7 (13.2%)	6 (12.2%)	1.000
Margin not free	3 (5.7%)	3 (6.1%)	1.000
ENE	5 (9.4%)	4 (8.2%)	1.000
Tumor size (cm), mean ± SD	2.6 ± 1.5	2.3 ± 1.4	0.278
Lymph node invasion			0.646
Positive	12 (22.6%)	14 (28.6%)	
Negative	41 (77.4%)	35 (71.4%)	
Pathological stage			0.629
I-II	36 (67.9%)	39 (79.6%)	
III-IV	17 (32.1%)	10 (20.4%)	
Death	17 (32.1%)	9 (18.4%)	0.174
Progressed	25 (47.2%)	11 (22.4%)	**0.016**

P-value is estimated using independent two-sampled t-test, chi-squared test or Fisher's exact test.

Supplementary Table S3. Comparison of the prediction ability of different protein location on overall mortality and disease-progressed.

Protein	Death			Progression		
	nu/mem (AUC)	cy (AUC)	*P*	nu/mem (AUC)	cy (AUC)	*P*
P16	0.471	0.485	0.697	0.543	0.544	0.962
RB1	0.554	0.500	0.359	0.518	0.500	0.744
EGFR	0.474	0.494	0.728	0.425	0.523	0.053
CDK6	0.489	0.481	0.893	0.496	0.508	0.839
PhosphoMet	0.426	0.500	0.179	0.469	0.500	0.552
POLB	0.570	0.583	0.883	0.495	0.529	0.684
SHC1	0.519	0.597	0.459	0.501	0.559	0.551
CDH3	0.453	0.423	0.609	0.460	0.469	0.878
STK17A	0.472	0.427	0.553	0.479	0.476	0.970
PIM1	0.465	0.443	0.744	0.485	0.476	0.889
FLNA	0.551	0.516	0.638	0.480	0.469	0.891
